# Rescue intracranial stenting for acute ischemic stroke after the failure of mechanical thrombectomy: A systematic review, meta-analysis, and trial sequential analysis

**DOI:** 10.3389/fneur.2023.1023089

**Published:** 2023-01-25

**Authors:** Junxiu Cai, Hai Xu, Rongzhou Xiao, Liping Hu, Ping Xu, Xianbin Guo, Yu Xie, Min Pan, Jie Tang, Qingtao Gong, Yan Liu, Rong Su, Jiahua Deng, Li Wang

**Affiliations:** ^1^Department of Neurology, Zigong Third People's Hospital, Zigong, China; ^2^Department of Neurology, Ziyang People's Hospital, Ziyang, China; ^3^Department of Radiology, Zigong Third People's Hospital, Zigong, China; ^4^Clinical Laboratory, Zigong Third People's Hospital, Zigong, China; ^5^Department of Neurology, Huili People's Hospital, Huili, China

**Keywords:** acute ischemic stroke, mechanical thrombectomy failure, rescue stenting, meta-analysis, trial sequential analysis

## Abstract

**Background:**

Intracranial rescue stenting (RS) might be an option for acute ischemic stroke after the failure of mechanical thrombectomy (MT). However, the findings were not consistent in previous systematic reviews, and whether the conclusion was supported by sufficient statistical power is unknown.

**Aim:**

To examine the effect of RS on acute ischemic stroke after the failure of MT with a systematic review, meta-analysis, and trial sequential analysis (TSA).

**Methods:**

We searched Ovid Medline, Embase, and the Cochrane Central Register of Controlled Trials (CENTRAL) from inception to 15 June 2022, without any language restriction. Studies assessing the effect of RS for acute ischemia stroke after MT failure were included. Two reviewers independently screened the retrieved articles, extracted data, and evaluated the quality of the included studies through the New Ottawa Scale (NOS). The primary outcome was the recanalization rate after RS. Secondary outcomes included modified Rankin Scale (mRS) at 3 months after stroke, symptomatic intracranial hemorrhage (sICH), and mortality rate. We synthesized the data through a random-effects model and performed a TSA analysis.

**Results:**

We included 15 studies (containing 1,595 participants) after screening 3,934 records. The pooled recanalization rate for rescue stenting was 82% (95% CI 77–87%). Compared with non-stenting, rescue stenting was associated with a higher proportion of patients with 0–2 mRS score (OR 3.96, 95% CI 2.69–5.84, *p* < 0.001) and a lower 90-day mortality rate (OR 0.46, 95% CI 0.32–0.65, *p* < 0.001), and stenting did not increase sICH rate (OR 0.63, 95% CI 0.39–1.04, *p* = 0.075). The TSA analysis showed that the meta-analysis of the mRS score had a sufficient sample size and statistical power.

**Conclusions:**

Our study showed that rescue stenting was effective and safe for patients with acute ischemia stroke who also had a failed MT, and this result was confirmed in a TSA analysis.

## Introduction

The prevalence and incidence of ischemic stroke are increasing because the global population ages, the absolute number of incidence of ischemic stroke increases from 4,309,356 in the year 1990 to 6,892,857 in the year 2013 (1). Stroke is now the second leading cause of death and a major cause of disability worldwide. The extent of collateral circulation in patients with ischemic stroke was closely related to their clinical outcomes. A good collateral circulation normally correlates with a good clinical outcome, and good collateral circulation has also been associated with the greater benefit of intravenous thrombolysis and endovascular treatment (2).

Mechanical thrombectomy (MT) is now becoming the first-line treatment option for reperfusion in patients with acute ischemic stroke, especially for those with contraindications for intravenous thrombolysis (3). Although promising, MT therapy still has a high rate of failure, which is estimated to be around 30% (4). Several approaches were proposed for patients with failed MT attempts, and glycoprotein IIb/IIIa inhibitors, balloon angioplasty, and rescue stenting are the three most frequently selected (5). Rescue stenting is of great interest to neurologists since it is a non-pharmacological therapy that could avoid the contraindications of pharmacological treatments and has a high rate of recanalization which enables rapid reperfusion. Previous systematic reviews have examined the effectiveness of rescue stenting for patients with acute ischemic stroke after MT failed, and the results showed that rescue stenting had favorable rates of recanalization and led to a better functional outcome than non-stenting treatments (6–9). However, owing to small sample sizes and the observation nature of the included studies, these reviews could not reach firm conclusions.

In the recent 2 years, studies that focused on the effectiveness of rescue stenting for patients with failed MT treatment were emerging (10–12). Among them, one study published in 2022 recruited 499 participants—the largest sample size today (12), and the study adopted the design of propensity score matching, which balanced the baseline characteristics between the stenting and non-stenting group and therefore generated a more robust result than previous studies did. The addition of these studies in a new systematic review with meta-analysis might further clarify whether rescue stenting is effective and safe for patients with acute ischemia stroke who had at least one failed MT.

Insufficient sample size and repeated significance testing are the major threats to the generation of robust results in meta-analyses. Trial sequential analysis (TSA), a statistical analysis method analog to interim analyses in randomized controlled trials, is believed to have better control over type-I and type-II errors in a meta-analysis (13). In addition, the TSA analysis can estimate the needed sample size in a meta-analysis for a pre-specified effect size (14).

Based on the grounds, we performed a systematic review with meta-analysis and TSA, aiming to examine whether rescue stenting after the failure of MT improves the outcomes in patients with acute ischemic stroke.

## Methods

A systematic review with meta-analysis was conducted to examine the effectiveness of rescue stenting after mechanical thrombectomy for acute ischemia stroke, which was conducted according to PRISMA (15). We acquired summary-level data from published literature, and ethical approvals were acquired in each original study.

### Literature search

We searched Ovid Medline, Embase, and the Cochrane Controlled Register of Trials (CENTRAL) from inception to 15 June 2022, setting no language restriction during the literature search. We searched the databases with the search strategies combining the following keywords: stroke, middle cerebral artery, thrombectomy, endovascular, clot retrieval, rescue stenting, and angioplasty. We read the reference lists of the previously published systematic reviews and meta-analyses, to check whether studies were missing from the literature search. We searched the website of the American Academy of Neurological Surgery (https://americanacademyns.org/), the European Association of Neurosurgical Societies (https://www.eans.org), and the American Association of Neurological Surgeons (https://www.aans.org/) for meeting abstracts and conference posters that reported studies of interest.

### Study screening

The inclusion criteria of this meta-analysis included: (1) participants with ischemia stroke who had at least one attempt of MT but failed [defined as modified Thrombolysis in Cerebral Infarction (mTICI) score ≤ 2b]; (2) participant's age > 18 years; (3) participants received rescue stenting after failed MT, and the type of stents and stenting procedure were not limited; (4) observational studies (case series, cohort studies) and experimental design (randomized controlled trials) were all included; (5) the control group being no rescue stenting or normal medical care; (6) studies that reported any of the following outcomes: recanalization rate, Modified Rankin Scale (mRS), assessment of symptomatic intracranial hemorrhage (sICH), and mortality rate.

The exclusion criteria included: (1) studies that recruited < 5 participants in the rescue stenting arm; (2) studies that reported the outcomes but with missing and insufficient data for analysis; (3) studies that were reported in the form of conference abstracts, research letters, or news reports.

The study screening was performed by one reviewer, and the results were checked and confirmed by another reviewer. The titles and abstracts of the retrieved articles were first screened, and the remained articles of interest were further searched for full-text copies. Disagreements in the study selection between the two reviewers were arbitrated by a third reviewer.

### Outcome measurements

The outcome measurements included recanalization rate, mRS score, sICH rate, and 90-day mortality. The recanalization rate was normally reported for the rescue stenting arm but not for the non-stenting arm, so our primary outcome was an mRS score from 0 to 2, a score range that is conventionally recognized as achieving a good outcome following stroke (16). The mRS is an ordinal scale that ranges from 0 (no symptoms at all) to 6 (death), and the score of 1 indicates no significant disability despite symptoms while the score of 2 indicates slight disability but able to look after own affairs without assistance (17). The sICH was considered the potentially harmful effect of stenting treatment, especially in the circumstance that antiplatelet medications should be used after the stenting procedure. We, therefore, assessed this outcome to evaluate the safety of rescue stenting. Previous studies demonstrated a decrease in 90-day mortality in patients receiving rescue stenting, so we assessed it as an efficacy outcome.

### Data extraction

Standardized forms, designed and entered through Excel software (Excel 2016), were used to extract data from the included studies. Two reviewers independently extracted the following information: the name of the first author, year of publication, the country where the studies were conducted, the total number of participants, study design, the use of propensity score matching analysis (yes or no), the type of control group, mean age, the proportion of participants using intravenous tPA, the proportion of participants with middle cerebral artery (MCA) occlusion, the type of rescue stenting, and the assessed outcomes. The reviewers also extracted outcome parameters (i.e., means, standard deviations, events, number of participants in the stenting or non-stenting arm) from the included studies, and they tried to contact the authors when the data needed for meta-analysis were not reported in the articles. A third reviewer checked and validated the extracted data, and passed the cleared data to a statistician.

### Assessment of study quality

The quality of the included studies was assessed by using the Newcastle-Ottawa Scale (NOS), which assesses the quality of study design in three domains: the selection of cohorts, the comparability between cohorts, and the assessed outcomes. Possible total points are four points for selection, two points for comparability, and three points for outcomes. A higher score on the NOS scale indicates a better study quality.

### Statistical analysis

We reviewed and summarized the recanalization rate of rescue stenting after mechanical thrombectomy for acute stroke. Owing to the lack of controls for this outcome, we performed a single-proportions meta-analysis to pool the proportions of recanalization reported in the included studies. This meta-analysis was performed with the use of the inverse-variance weighted method (18), and the generalized linear mixed model was adopted for analysis to test the robustness of the findings.

For the outcome of 90-day mRS (0–2), sICH, and 90-day mortality, we first calculated the odds ratio (OR) of rescue stenting vs. non-stenting in these outcomes, and we secondly pooled the ORs using a fixed-effect model when the *I*^2^ value was under 50%. The 95% confidence intervals (95% CIs) of the pooled effect sizes were estimated, and the *p*-values of the comparisons were also provided. The heterogeneity of the meta-analysis was estimated by using the Cochran *Q*-test, and the degree of the heterogeneity was estimated by using the *I*^2^ value—with a cut-off point of 50% to determine whether there was significant heterogeneity. The meta-analysis was conducted in the R environment (R 4.0.1, meta package).

We performed a TSA analysis on the outcome of 90-day mRS, since it is the most commonly used measurement for the functional outcome of patients with stroke. The TSA analysis investigates the type-I error in the aggregated result of the meta-analysis—repeated significance testing increases the risk of type-I error. We re-adjusted the significance level by using the O'Brien-Flemming α-spending function, and the type-I error was controlled at the level of 0.05 while the type-II error was controlled at 0.2. We plotted the cumulative *Z*-curve of the meta-analysis to define sequential boundaries to infer the levels of type-I and type-II errors, calculate the required information size (RIS), and determine whether further trials in the field is needed—when the total sample size of recruited participants exceeds the RIS, further studies are not required. The TSA analysis was conducted by using the TSA software (V. 0.9.5.10).

## Results

### Study characteristics

We retrieved 3,934 records from the three databases: 1,587 from Medline, 1,988 from Embase, and 359 from CENTRAL. A total of 15 studies were finally included (10–12, 19–30). Before the full-text screening, we excluded 1,634 duplicates, 1,288 records because of reviews, abstracts only, or conference papers without detailed information, 527 records that reported the effect of mechanical thrombectomy, and 368 records that were not relevant to stenting treatment. One hundred and seventeen records were sought for retrieval, and 15 of them were unavailable for full-text copies. In the full-text assessment, 13 records were excluded for a sample size < 5, 28 records for reviews, 36 records for mechanism studies, and 10 records for meta-analysis, with no available data or no intended outcomes. The process and flowchart of screening were shown in the Supplementary Figure 1.

The included 15 studies recruited 1,595 participants, and these studies were published from the year 2015–2022. Six of the studies were from South Korea, three from China, two from the USA, two from Italy, and the rest two from Spain and Sweden. The study with the largest sample size was from the USA, recruiting 499 participants. Five studies adopted a prospective design, and one of the five studies adopted a multicenter design; the rest 10 studies were with retrospective design. Seven studies had non-stenting arms as the control group. The mean age of the participants ranged from 61.4 to 70.1 years. All the studies assessed the mRS score and used the score of 0–2 as the indicator of a good outcome. The other information, the proportion of patients who used intravenous tPA, the proportion of patients with middle artery occlusion, and the assessed outcomes were shown in Table 1. Eight studies were rated six points by using the NOS scale, three were rated seven points, two were rated eight points, and the rest two were rated nine points.

**Table 1:** Characteristics of the included trials.

**References**	**Country**	**Sample size**	**Study design**	**Propensity score matching**	**Control group**	**Mean age**	**IV tPA (%)**	**MCA occlusion**	**Rescue stenting type**	**Assessed outcomes**	**Nos** **score**
Kasab et al. (19)	USA	36	Retrospective single-center cohort study	No	No	66.4 (14.1)	NA	NA	Wingspan, Precise, Enterprise	Recanalization rate (mTICI 2b-3); revascularization time; mean procedural time; mRS; postprocedural complications	6
Baek et al. (20)	South Korea	45	Retrospective single-center cohort study	No	Non-stenting	70.1 (11.1)	31.1	55.6	Solitaire AB/FR, Wingspan	Recanalization rate (mTICI 2b-3); mRS; cerebral herniation rate; sICH; mortality rate	7
Baracchini et al. (21)	Italy	109	Prospective single-center cohort study	No	Non-stenting	65 (15.3)	NA	80.4	Solitaire AB	Recanalization rate (mTICI 2b-3); mRS; sICH; mortality rate	7
Chang et al. (22)	South Korea	148	Retrospective multicener-center cohort study	No	Non-stenting	66.6 (13.7)	49.5	63.5	Solitaire AB, Wingspan, Enterprise, balloon expandable	Recanalization rate; mRS; sICH; mortality rate	8
Cornelissen et al. (23)	Sweden	26	Retrospective single-center cohort study	No	Non-stenting	67.3 (9.5)	34.6	NA	Enterprise, Solitaire	mRS; mortality rate	7
Delgado Acosta et al. (24)	Spain	42	Retrospective single-center cohort study	No	No	61 (53–72)	NA	NA	Enterprise	Recanalization rate; mRS; sICH; mortality rate	6
Kim et al. (25)	South Korea	46	Retrospective single-center cohort study	No	No	66 (58–75)	47.1	56.5	Wingspan	Recanalization rate; mRS; sICH; mortality rate	6
Nappini et al. (26)	Italy	17	Retrospective single-center cohort study	No	No	62 (37–80)	47	41.1	Solitaire AB	Recanalization rate; mRS; sICH; mortality rate	6
Peng et al. (10)	China	132	Retrospective multicenter case-control study	Yes	Non-stenting	66 (55–76)	31.8	NA	Solitaire, Stryker	Recanalization rate; mRS; sICH; mortality rate	9
Seo et al. (27)	South Korea	10	Prospective single-center cohort study	No	No	62.5 (11.3)	10	40	Wingspan	Recanalization rate; mRS	6
Woo et al. (28)	South Korea	27	Retrospective single-center cohort study	No	No	NA	NA	NA	Solitaire FR	Recanalization rate; mRS; sICH; mortality rate	6
Yoon et al. (29)	South Korea	172	Retrospective single-center cohort study	No	No	69.1 (9.5)	50.6	58.7	Wingspan	Recanalization rate; mRS; sICH; mortality rate	6
Zhou et al. (30)	China	193	Prospective single-center cohort study	No	No	63 (12.1)	23.3	NA	Solitaire, Apollo, Enterprise, Wingspan, Neuroform	Recanalization rate; time from groin puncture to recanalization; mRS; sICH; mortality rate	6
Luo et al. (11)	China	93	Prospective single-center cohort study	No	Non-stenting	61.4 (12)	17.2	NA	NA	Recanalization rate; mRS; sICH; mortality rate	8
Mohammaden et al. (12)	USA	499	Prospective multicenter case-control study	Yes	Non-stenting	65.2 (14.9)	29	67.3		mRS; sICH; mortality rate	9

mRS, modified Rankin Scale; NOS, Newcastle-Ottawa scale; sICH, symptomatic intracranial hemorrhage.

### Recanalization rate

**Figure 1:**
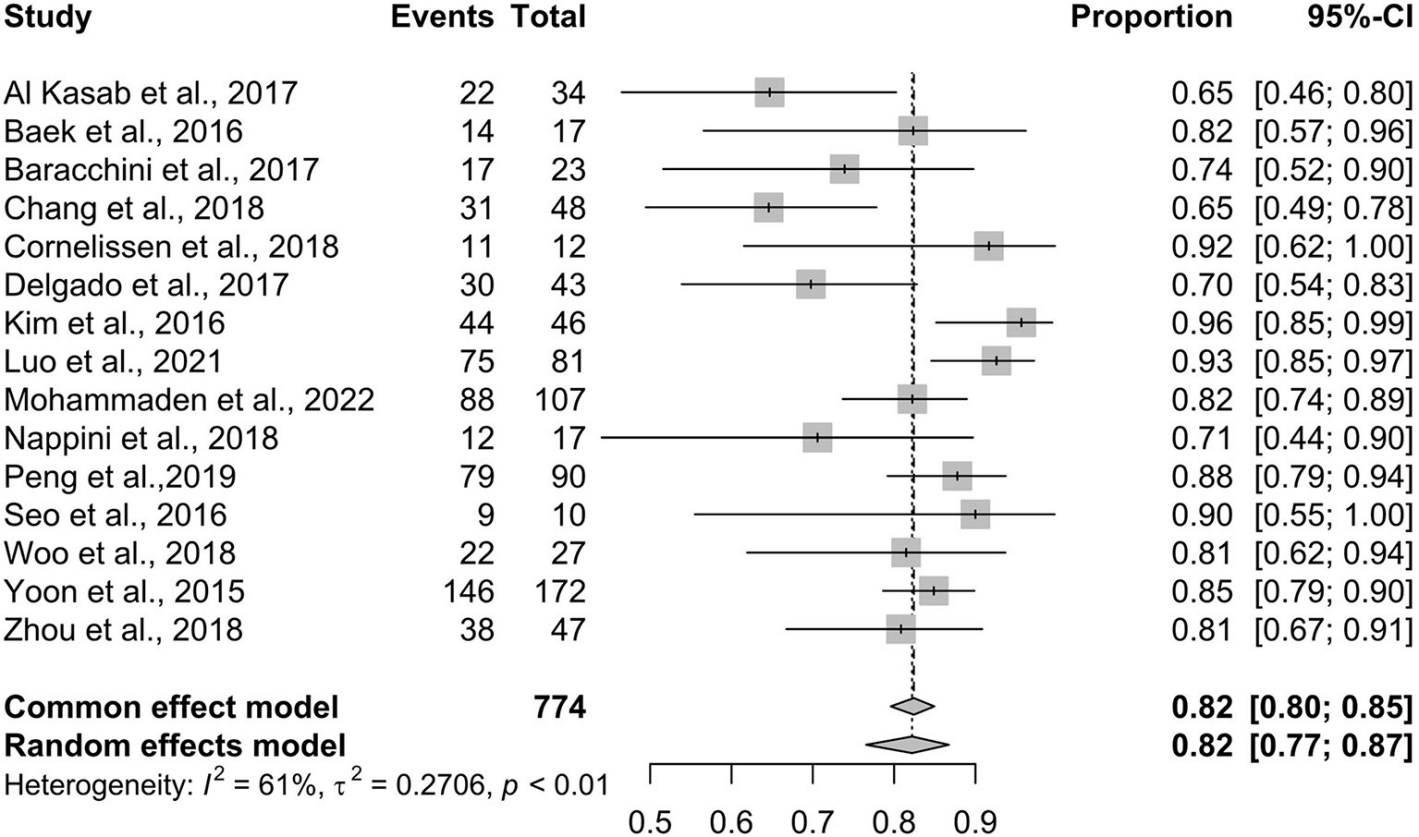
Recanalization rate.

Figure 1 shows the pooled result of the recanalization rate. Fifteen studies recruiting 774 participants were included, and the results showed a recanalization rate of 82% (95% CI 77–87%). A large and significant heterogeneity was noticed in the analysis (*I*^2^ = 61%, *p* < 0.01); the lowest recanalization rate was 65% (19) while the highest rate was 96% (25).

### 90-days mRS

**Figure 2:**
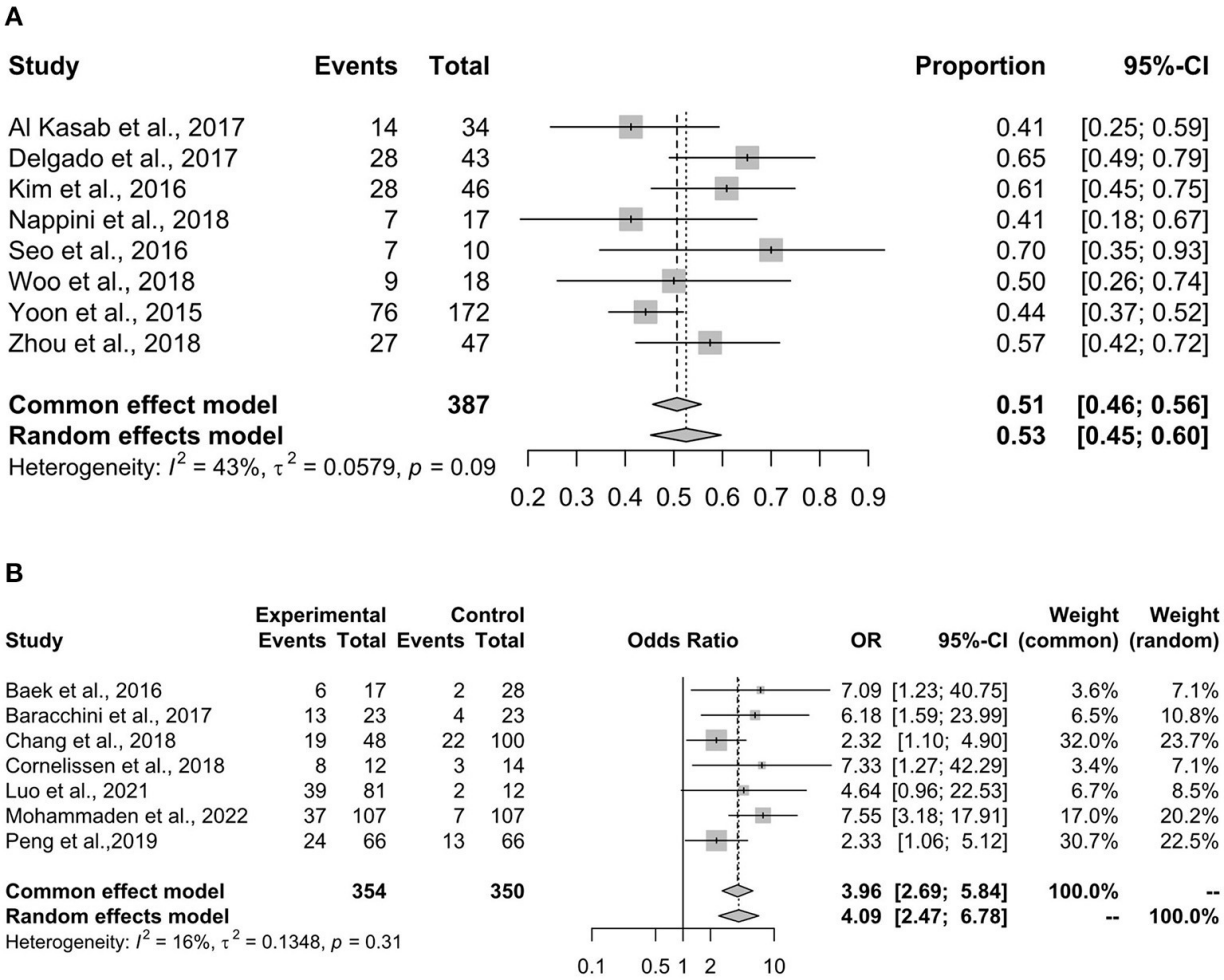
The mRS score assessment. The mRS score of 0–2 indicates a good outcome of patients with ischemia stroke. **(A)** Summarizes the proportion of patients with 0–2 mRS scores, using data from cohorts without control groups. **(B)** Shows the comparison of stenting vs. non-stenting in the proportion of patients with 0–2 mRS score, and a higher OR indicates a better result of the stenting arm. mRS, modified Rankin Scale; OR, odds ratio.

Figure 2A shows the pooled result of the proportion of patients with 0–2 mRS scores, which indicates a good outcome. The fixed-effects model estimated a proportion of 51% (95% CI 46–56%) of the participants with 0–2 mRS. The heterogeneity was small and insignificant in this analysis (*I*^2^ = 43%, *p* = 0.09).

Figure 2B shows the comparison of rescue stenting vs. non-stenting in achieving the outcome of 0–2 mRS. The results showed that stenting had a significantly higher success rate in achieving 0–2 mRS (OR 3.96, 95% CI 2.69–5.84, *p* < 0.001), and the fixed-effect model was consistent with the random-effect model. A small and insignificant heterogeneity was noted in the analysis (*I*^2^ = 16%, *p* = 0.31).

### sICH

**Figure 3:**
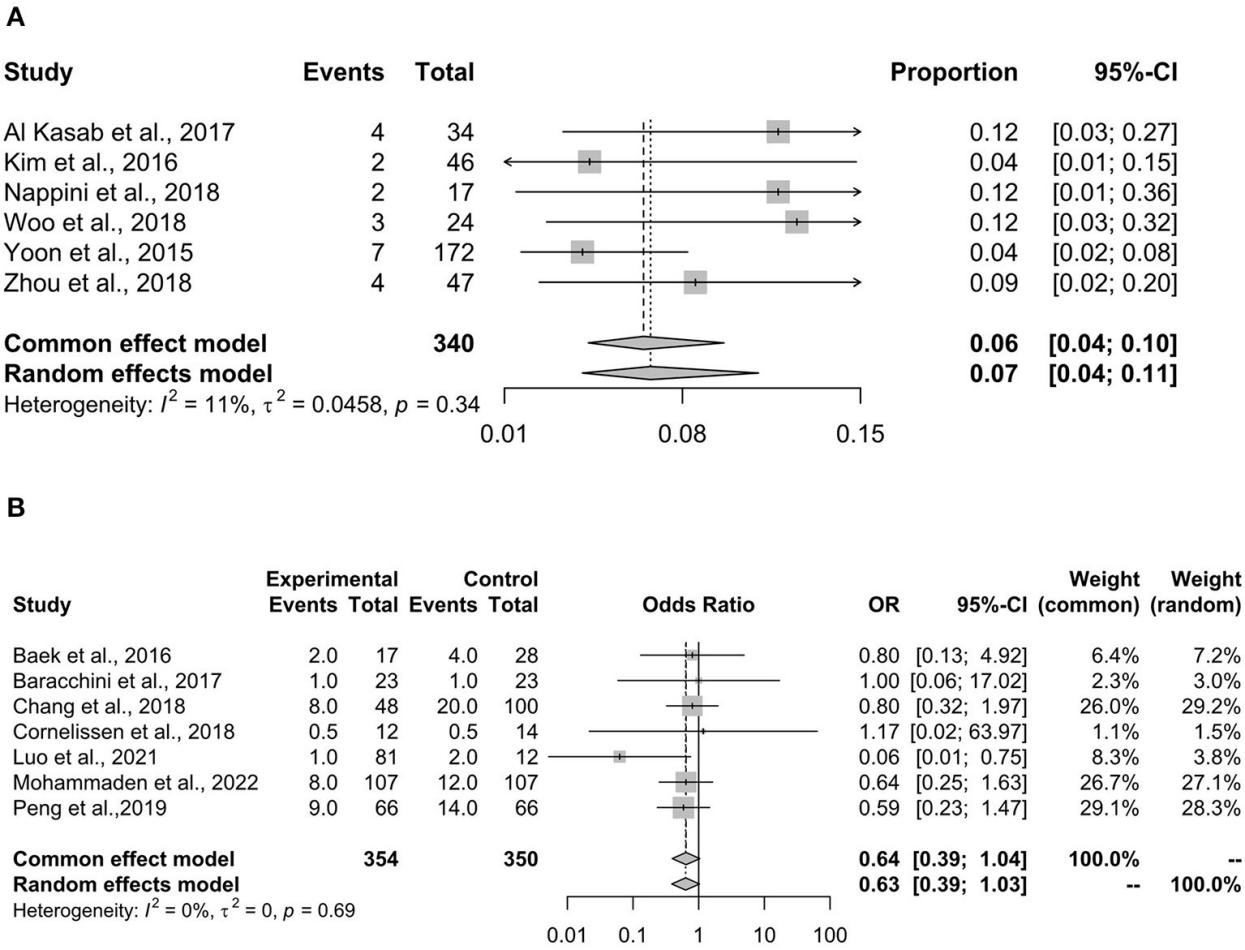
The proportion of patients with sICH. sICH, symptomatic intracranial hemorrhage. sICH is a negative outcome for patients receiving rescue stenting. **(A)** Summarizes the proportion of patients developing sICH after receiving stenting. **(B)** Shows the comparison of stenting with non-stenting in the proportion of patients developing sICH, and a lower OR indicates a better result for the stenting arm. OR, odds ratio.

Figure 3A shows the synthesized result of the proportion of patients who developed sICH after rescue stenting. The result showed that the incidence of sICH was 6% (95% CI 4–11%). The results were consistent between the fixed-effects model and the random-effects model. The heterogeneity in this analysis was small (*I*^2^ = 11%) and insignificant (*p* = 0.34).

Figure 3B shows the comparison of stenting vs. non-stenting in the proportion of patients with sICH. The results showed that stenting had a lower but not statistically significant proportion of sICH compared with non-stenting (OR 0.63, 95% CI 0.39–1.04, *p* = 0.075). The fixed-effects and random-effects models showed consistent results. No heterogeneity was detected in this analysis (*I*^2^ = 0%, *p* = 0.69).

### 90-days mortality

**Figure 4:**
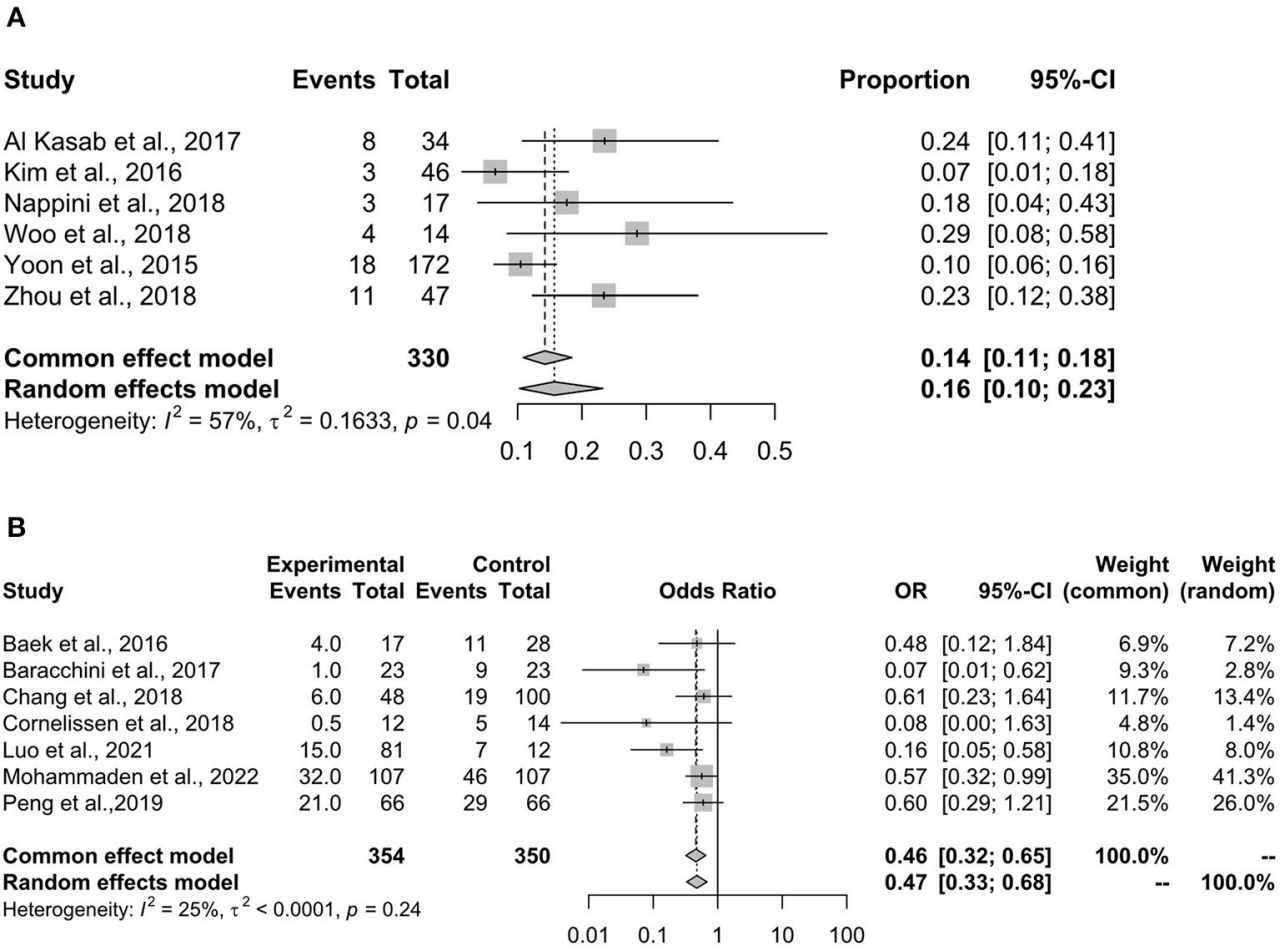
90-day mortality. 90-day mortality is a negative outcome for patients receiving rescue stenting. **(A)** Summarizes the proportion of 90-day mortality. **(B)** Shows the comparison of stenting with non-stenting in the proportion of 90-day mortality, and a lower OR indicates a better result for the stenting arm. OR, odds ratio.

Figure 4A shows the pooled result of the 90-day mortality rate, which showed a synthesized mortality rate of 16% (95% CI 10–23%). The heterogeneity was large and significant (*I*^2^ = 57%, *p* = 0.04), so the result of the random-effects model was adopted.

Figure 4B shows the comparison of stenting vs. non-stenting in the 90-day mortality rate, which showed that stenting was associated with a significantly lower mortality rate when compared with non-stenting (OR 0.46, 95% CI 0.32–0.65, *p* < 0.001). A small and insignificant heterogeneity was found in the analysis (*I*^2^ = 25%, *p* = 0.24).

### TSA analysis

**Figure 5:**
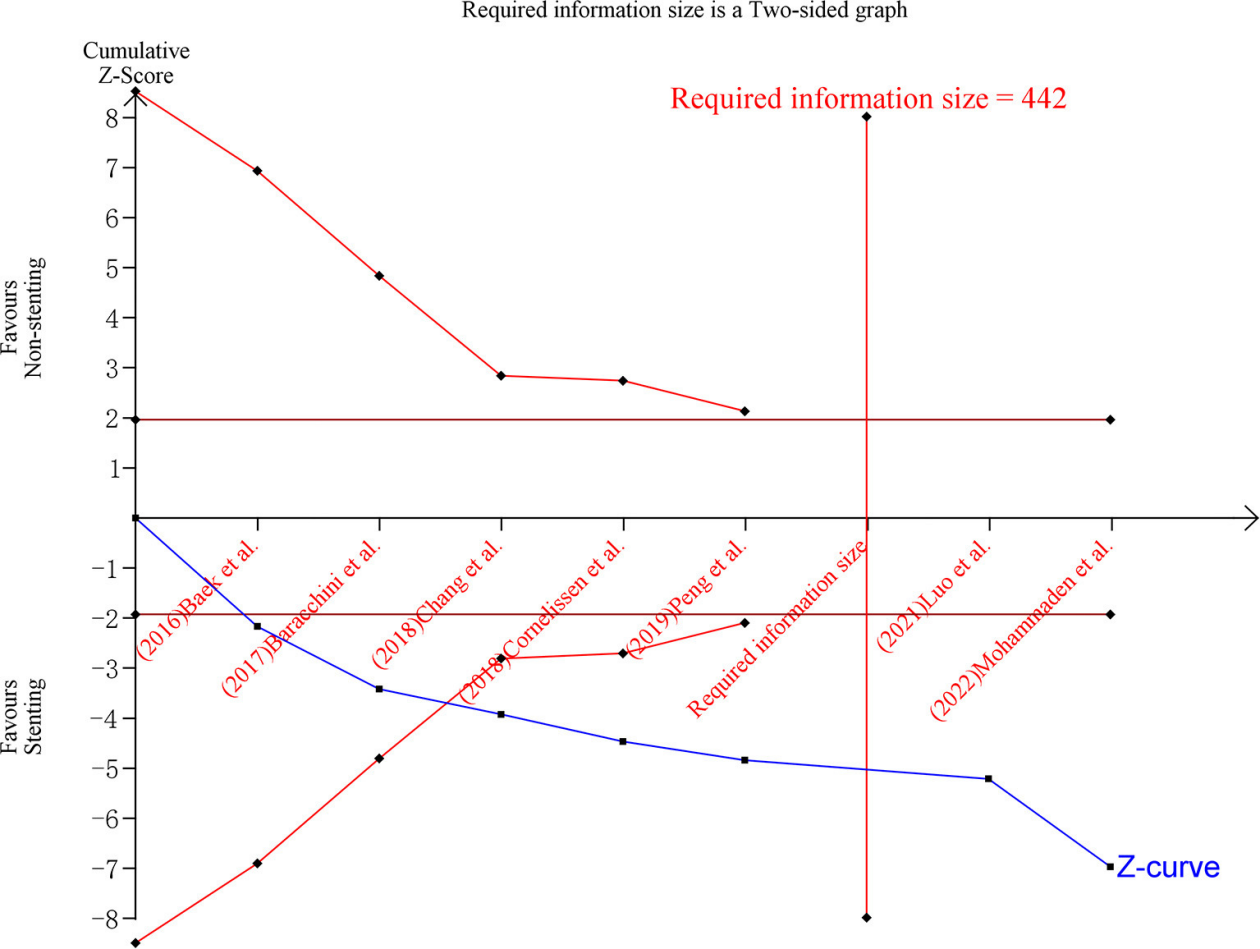
TSA analysis on the mRS score. mRS score of 0–2 is a positive outcome for patients receiving rescue stenting. The *x*-axis ticks refer to the studies added sequentially into the analysis. The blue curve was the adjusted *Z*-curve, and it crossed the dark-red horizontal line (conventional boundary for α = 0.05). The vertical red line shows the required information size—the needed sample size for a robust result with sufficient statistical power. The analysis included 704 participants, which exceed the required information size (*n* = 442). mRS, modified Rankin Scale; TSA, trial sequential analysis.

Figure 5 shows the result of TSA analysis on the comparison of stenting vs. non-stenting in the outcome of achieving a 0–2 mRS score. Considering a 50% rate of 0–2 mRS score in the stenting arm and a 35% rate in the non-stenting arm—resulting in a ratio difference of 15%, a type-I error of 0.05, and a type-II error of 0.2, the required information size would be 442 participants. The sample size of our meta-analysis was 704 participants, which exceeds the required information size. Figure 5 also shows that the difference between stenting and non-stenting in the proportion of 0–2 mRS score was evident since the cumulative *Z* curve had crossed conventional boundaries—the result was in favor of the stenting arm.

## Discussion

Whether rescue stenting should be recommended for patients with acute stroke having a failed mechanical thrombectomy is still controversial. We performed a systematic review to evaluate current evidence and a meta-analysis incorporating TSA analysis to examine the benefit of rescue stenting and estimate whether the primary result has a sufficient sample size. First, we found that stenting had a high recanalization rate and was beneficial for patients after the failure of mechanical thrombectomy. The pooled recanalization rate of stenting was 82%; the proportion of patients with a 0–2 mRS score was 51% after receiving stenting, and patients receiving stenting had a significantly higher rate of achieving a 0–2 mRS score—indicating a good outcome of functional capability. The other outcomes—sICH rate and 90-day mortality rate—supported the use of stenting, which was associated with a significantly lower 90-day mortality but did not cause a higher rate of sICH. Second, we confirmed that current evidence is of sufficient power to detect a 15% difference in the proportion of patients achieving a 0–2 mRS score. This finding indicated that the advantage of stenting is confirmed when compared with non-stenting, since the ratio difference in achieving a 0–2 mRS score between stenting and non-stenting exceeded 15% (a pooled OR of 3.96 and an estimated ratio difference of 31% in our meta-analysis). To the best of knowledge, our study was the first to adopt the TSA analysis to confirm whether rescue intracranial stenting was effective for acute ischemic stroke after the failure of MT.

The results of our meta-analysis were consistent with the previously published systematic reviews with meta-analysis (6–9). These systematic reviews concluded that rescue stenting might be an effective treatment for patients who had failed MT procedures. However, all these reviews mentioned the same limitations—the small sample sizes and the observational design of the included studies. After the publication of these reviews, two studies with larger sample size and with a matched-analysis design to balance the baseline characteristics were published (10, 12). One study published in 2022 had the largest sample size on this topic (12), and it adopted a propensity score matching analysis—leading to more balanced baseline parameters in the stenting and non-stenting arms, which provided more accurate estimates than previous studies. We therefore performed a TSA analysis, which had not been studied in previous systematic reviews, to clarify whether current evidence had a sufficient sample size and statistical power. The TSA model was developed to address statistical problems that arise with multiplicity due to repeated significance testing (31). One study reported that a cumulative chance of a type-I error became 8, 14, 25, and 37% when the statistical hypothesis was repeatedly tested for two, five, twenty, and 100, respectively (13). In that case, our meta-analysis would have the risk of type-I error increasing to at least 14% if we did not perform a TSA to confirm the main findings. Our TSA analysis showed that, for the proportion of patients achieving a 0–2 mRS score, the results of the meta-analysis had a sufficient sample size and statistical power (80%) to reject a null hypothesis at the level of 0.05, which confirmed the robustness of results.

Several adjunct options for failed MT were proposed, including antiplatelet glycoprotein IIb/IIIa inhibitors, intracranial angioplasty, and rescue stenting (5). Glycoprotein IIb/IIIa inhibitors were normally used as adjunctive salvage interventions for early vessel reocclusion. However, it was not recommended in the management of acute ischemia stroke, since it was associated with a significant risk of intracranial hemorrhage with no evidence of any reduction in death or disability in survivors (32). Intracranial angioplasty, performed with the expansion of a balloon, was normally followed by stenting to prevent vessel reocclusion after reperfusion was achieved. Intracranial angioplasty with stenting was more frequently selected than glycoprotein IIb/IIIa inhibitors alone, according to a north American cohort (5).

The rescue stenting requires antiplatelet medications to prevent in-stent thrombosis and vessel reocclusion. Antiplatelet medications may increase the probability of sICH, especially in patients with large infarct volumes. Our meta-analysis showed that stenting did not increase the risk of sICH when compared with non-stenting (OR 0.63, 95% CI 0.39–1.04). The finding was consistent with previous systematic reviews (7, 9) and a recent large-scale cohort (12). This finding should be further confirmed in randomized controlled trials, since participants who chose non-stenting treatments might be at higher risk of intracranial hemorrhage—for example, they might choose a higher dose of antiplatelet medication to manage reocclusion or deterioration of stenosis, which might lead to a higher risk of intracranial hemorrhage.

Our study had limitations. First, the observational design of the included studies would be affected by confounding factors. Although the recent large-scale matched analysis provided a more robust estimation of the effect of rescue stenting for this condition, the results were still under the risk of confounding bias. The matched analysis, normally propensity score matching analysis, could only adjust for known and measured factors. Randomized controlled trials are still the best solution for unmeasured factors that might cause bias in estimation. Second, the follow-up period is short. In most studies, the mRS score and mortality rate were only assessed for 90 days. In future studies, a follow-up period longer than 1 year might provide essential information for patients and clinicians in making their decision on whether permanent stenting should be preferred. Third, owing to the limited number of the includes studies and limited baseline information, we did not conduct subgroup analysis concerning region, study design or tPA factors.

In conclusion, our meta-analysis showed that rescue stenting was effective and safe for patients with ischemia stroke who also had a failed MT, and this result was confirmed in a TSA analysis—showing that the analysis had a sufficient sample size and statistical power for the mRS outcome.

## Data availability statement

The original contributions presented in the study are included in the article/Supplementary material, further inquiries can be directed to the corresponding authors.

## Author contributions

LW and JD proposed the conception and designed the protocol and critically reviewed or revised the manuscript for important intellectual content. JC and HX managed the study, acquired data, interpreted the results, and drafted the manuscript. RX and LH managed the study and performed the statistical analyses. PX, XG, YX, MP, JT, QG, YL, and RS supervised the data analysis, provided the interpretation of results, and contributed to the drafting and critical review of the manuscript. All authors approved the final draft.
