# Supplementary material for: Rescue intracranial stenting for acute ischemic stroke after the failure of mechanical thrombectomy: A systematic review, meta-analysis, and trial sequential analysis
Source: Front Neurol. 2023 Jan 25;14:1023089. doi: 10.3389/fneur.2023.1023089 (PMC9905111; doi:10.3389/fneur.2023.1023089)
Supplement: Supplementary file 1 [file Data_Sheet_1.docx]

**Supplementary files**

**Supplementary table 1. Search strategy in OVID Medline**

| No. | Search terms |
| --- | --- |
| 1 | randomised controlled trial.pt. |
| 2 | randomized controlled trial.pt. |
| 3 | controlled clinical trial.pt. |
| 4 | randomized.ab. |
| 5 | randomised.ab. |
| 6 | randomly.ab. |
| 7 | exp Cohort Studies/ |
| 8 | cohort studies.pt. |
| 9 | cohort.ab. |
| 10 | or/1-10 |
| 11 | limit 10 to humans |
| 12 | exp Stroke/ |
| 13 | exp Middle Cerebral Artery/ |
| 14 | exp Carotid Stenosis/ |
| 15 | ischemia stroke.ti,ab. |
| 16 | stenosis. ti,ab. |
| 17 | or/12-16 |
| 18 | thrombectomy. ti,ab. |
| 19 | endovascular. ti,ab. |
| 20 | clot retrieval. ti,ab. |
| 21 | rescue stenting. ti,ab. |
| 22 | angioplasty. ti,ab. |
| 23 | stent retrievers.ti,ab. |
| 24 | or/18-23 |
| 25 | 11 AND 17 AND 24 |

**Supplementary table 2. Search strategy in Embase**

| No. | Search terms |
| --- | --- |
| 1 | ‘randomized Controlled Trial’/exp |
| 2 | ‘randomized Controlled Trials as Topic’/exp |
| 3 | ‘randomized controlled trial’:ab,ti |
| 4 | ‘controlled clinical trial’/exp |
| 5 | ‘controlled clinical trial’:ab,ti |
| 6 | ‘Cohort Studies’/exp |
| 7 | ‘cohort’:ab,ti |
| 8 | (#1 OR #2 OR #3 OR #4 OR #5 OR #6 OR #7) AND [humans]/lim |
| 9 | ‘Stroke’/exp |
| 10 | ‘Middle Cerebral Artery’/exp |
| 11 | ‘Carotid Stenosis’/exp |
| 12 | ‘ischemia stroke’:ab,ti |
| 13 | ‘stenosis’:ab,ti |
| 14 | #9 OR #10 OR #11 OR #12 OR #13 |
| 15 | ‘thrombectomy’:ab,ti |
| 16 | ‘endovascular’:ab,ti |
| 17 | ‘clot retrieval’:ab,ti |
| 18 | ‘rescue stenting’:ab,ti |
| 19 | ‘angioplasty’:ab,ti |
| 20 | ‘stent retrievers’:ab,ti |
| 21 | #15 OR #16 OR #17 OR #18 OR #19 OR #20 |
| 22 | #8 AND #14 AND #21 |

**Supplementary table 3. Search strategy in CENTRAL**

| No. | Search terms |
| --- | --- |
| 1 | (randomised controlled trial):ti,ab,kw |
| 2 | (cohort):ti,ab,kw |
| 3 | #2 or #3 |
| 4 | (stroke):ti,ab,kw |
| 5 | (middle cerebral artery) :ti,ab,kw |
| 6 | (ischemia stroke):ti,ab,kw |
| 7 | (stenosis):ti,ab,kw |
| 8 | #4 or #5 or #6 or #7 |
| 9 | (thrombectomy):ti,ab,kw |
| 10 | (endovascular):ti,ab,kw |
| 11 | (clot retrieval):ti,ab,kw |
| 12 | (rescue stenting):ti,ab,kw |
| 13 | (angioplasty):ti,ab,kw |
| 14 | (stent retrievers) :ti,ab,kw |
| 15 | #9 or #10 or #11 or #12 or #13 or #14 |
| 16 | #3 and #8 and #15 |

Abbreviation: CENTRAL, Cochrane Central Register of Controlled Trials.

**Supplementary figure 1. flow chart**

**
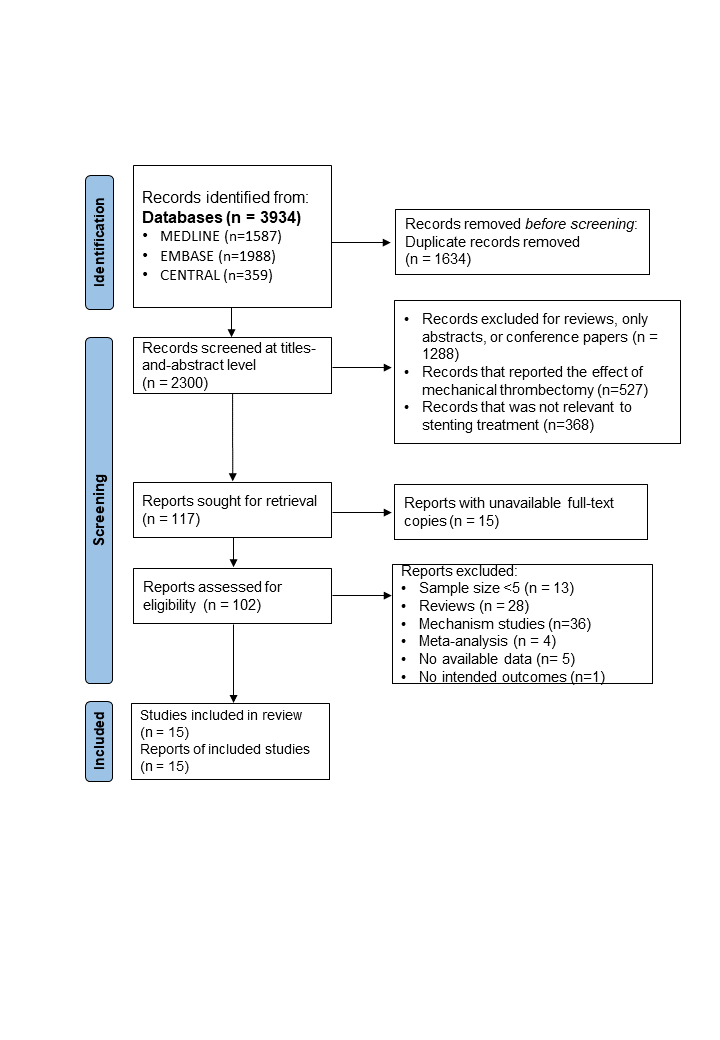
**
